# Editorial: Cognitive Impairment and Inflammation in Old Age and the Role of Modifiable Risk Factors of Neurocognitive Disorders

**DOI:** 10.3389/fpsyt.2021.784134

**Published:** 2021-11-26

**Authors:** Érico Castro-Costa, Breno S. Diniz, Sergio L. Blay

**Affiliations:** ^1^Center for Studies in Public Health and Aging, René Rachou Institute, Oswaldo Cruz Foundation, Belo Horizonte, Brazil; ^2^The University of Connecticut Health Center, Farmington, CT, United States; ^3^Department of Psychiatry, Federal University of São Paulo, São Paulo, Brazil

**Keywords:** brain aging, cognitive aging, cognitive impairment, inflammation, old age

Aging leads to a progressive decrease in the biological and functional integrity of several human parts of the body associated with augmented risk of disease and, eventually, susceptibility to death. This decline is a substantial threat for the leading human illness, encompassing cardiovascular disorders, cancer, diabetes, and psychiatric disorders (1).

So far, many anatomical and functional modifications linked to normal brain aging have been described and revealed a brain mass reduction of 2–3% per decade after the age of 50 and ~10% in older people with 80 years or older, when compared to young adults (2). However, cognitive decline differs substantially during aging, alternating from usual age-related modification to dementia (3).

*Cognitive aging* is a complex, multifactorial concept involving biological mechanisms such as immunosenescence, vascular aging, and brain aging. It is indicated by memory loss, other mental processes, changes in attitudes, disabilities to live independently (4). Additionally, it is a broad concept that comprises more than 100 diseases leading to dementia that has in common a progressive reduction in cognitive performance in old age. However, cognitive impairment could also appear across other psychiatric disorders aggravating their course and enormously compromising those patients with aging (5).

Recently, some researchers suggested an association between inflammatory processes and cognition. They have highlighted a close relationship between certain aspects of the immune system, impaired function of the blood-brain barrier, and changes in the functionally integrated network formed by neuron and vascular cells, leading to the release of reactive oxygen species (6). Additionally, another review discussed the importance of studying the effect of vascular changes, neuroinflammation, and Lewy body pathology on the cognitive decline of Alzheimer's disease (7). Finally, another research argued that Alzheimer's disease and vascular dementia might have common risk factors such as stroke that is involved in numerous cellular and molecular changes that comprise inflammation, mitochondrial malfunction, oxidative stress, vascular alterations, and significant modifications in brain proteins in both conditions (8).

Improved knowledge of the role of inflammation in the brain and cognitive brain aging is essential for emerging treatments to avoid or postpone age-related cognitive decline, therefore prolonging and improving health and quality of life in the following years. Several different pathways related to neuroinflammation have been connected in cognitive impairment and dementia.

This Research Topic included four articles that examined the clinical, immunological, neurotrophic, and metabolomic relationships with cognition. In the first article, Hansen N. et al. identified (in a case report) serum autoantibodies against the flotillin 1/2 complex that has never been reported in dementia patients and might have a potential role in autoantibody-associated psychiatric syndromes (9) and autoimmune dementia (10). While in the second paper, Huo et al. demonstrated for the first time that lower Brain-Derived Neurotrophic Factor (BDNF) was related to cognitive disturbances, particularly with attention dimension but not with psychotic symptoms in older schizophrenic patients. Therefore, it is possible to indicate BDNF as a reliable biomarker of cognitive function in old schizophrenic patients.

In addition, Lu et al. proposed, in the third study, the use of metabolomics that has potential understanding in the biological mechanism related to cognition impairment and might also be more sensitive and specific for diagnosing perioperative cognitive dysfunction (POCD). Furthermore, Hansen E. O. et al. validated a novel precise and low-cost test for assessing Alzheimer's biomarkers in cerebrospinal fluid that seems an ensuring instrument, particularly for low-and-middle-income countries, in the closing analysis.

Finally, we also included the study of Suehiro et al., which reported the possibility of Clerambault's syndrome during the early stages of dementia with Lewy bodies and might be essential for diagnosing this condition.

This significant increase in the proportion of older adults worldwide poses several life-threatening concerns for all countries, including cognitive aging, augmented numbers of people with dementia, and loss of independence. Therefore, we hope that this Research Topic might improve understanding of the role of inflammation in cognition in old age.

## Author Contributions

ÉC-C: concept, design, preparation, and critical revision of the manuscript. BSD and SLB: preparation and critical revision of the manuscript. All authors have read and approved the final version of the manuscript.

## Conflict of Interest

The authors declare that the research was conducted in the absence of any commercial or financial relationships that could be construed as a potential conflict of interest.

## Publisher's Note

All claims expressed in this article are solely those of the authors and do not necessarily represent those of their affiliated organizations, or those of the publisher, the editors and the reviewers. Any product that may be evaluated in this article, or claim that may be made by its manufacturer, is not guaranteed or endorsed by the publisher.

## References

[1] López-Otín C Blasco MA Partridge L Serrano M Kroemer G. The hallmarks of aging. Cell. (2013) 153:1194–217. 10.1016/j.cell.2013.05.039 23746838 PMC3836174

[2] Drachman DA. Aging of the brain, entropy, and Alzheimer's disease. Neurology. (2006) 67:1340–52. 10.1212/01.wnl.0000240127.89601.83 17060558

[3] Deary IJ Corley J Gow AJ Harris SE Houlihan LM Marioni RE. Age-associated cognitive decline. Br Med Bull. (2009) 92:135–52. 10.1093/bmb/ldp033 19776035

[4] Cunningham C Hennessy E. Co-morbidity and systemic inflammation as drivers of cognitive decline: new experimental models adopting a broader paradigm in dementia research. Alzheimer's Res Ther. (2015) 7:33. 10.1186/s13195-015-0117-2 25802557 PMC4369837

[5] Fourrier C Singhal G Baune BT. Neuroinflammation and cognition across psychiatric conditions. CNS Spectr. (2019) 24:4–15. 10.1017/S1092852918001499 30714555

[6] Tangestani Fard M Stough C. A review and hypothesized model of the mechanisms that underpin the relationship between inflammation and cognition in the elderly. Front Aging Neurosci. (2019) 11:56. 10.3389/fnagi.2019.00056 30930767 PMC6425084

[7] Gauthier S Zhang H Ng KP Pascoal TA Rosa-Neto P. Impact of the biological definition of Alzheimer's disease using amyloid, tau, and neurodegeneration (ATN): what about the role of vascular changes, inflammation, Lewy body pathology? Transl Neurodegener. (2018) 7:12. 10.1186/s40035-018-0117-9 29876101 PMC5977549

[8] Vijayan M Reddy PH. Stroke, vascular dementia, and Alzheimer's disease: molecular links. J Alzheimer's Dis. (2016) 54:427–43. 10.3233/JAD-160527 27567871 PMC5793908

[9] Endres D Maier V Leypoldt F Wandinger KP Lennox B Pollak TA. Autoantibody-associated psychiatric syndromes: a systematic literature review resulting in 145 cases. Psychol Med. (2020) 2020:1–12. 10.1017/S0033291720002895 32892761 PMC9069350

[10] Gibson LL McKeever A Cullen AE Nicholson TR Aarsland D Zandi MS. Neuronal surface autoantibodies in dementia: a systematic review and meta-analysis. J Neurol. (2021) 268:2769–79. 10.1007/s00415-020-09825-0 32306172 PMC8289796

